# Suicide planning type interventions as an evidence based alternative for no-suicide contracts

**DOI:** 10.1192/j.eurpsy.2024.1619

**Published:** 2024-08-27

**Authors:** A. Garbacka, M. Bzowska

**Affiliations:** ^1^Child and Adolescen Psychiatry, Uzdrowisko Konstancin Zdroj, Konstancin-Jeziorna; ^2^Psychiatry Private Practice, Warsaw, Poland

## Abstract

**Introduction:**

Suicidality is a common concern in psychiatric patients and one of the leading causes of death in adolescents and young adults. (*Adolescent health.* (*2019, November 26) WHO). S*ome mental health professionals engage in a no-suicide contract with their patients. In this type of intervention, the patient usually agrees to not harm or kill himself/herself. There is an increasing body of evidence to support brief interventions, such as group of safety planning-type interventions (SPTIs) *(McCabe et al. MC Psychiatry, 2018, May 3; 18(1)).* Safety planning is derived from cognitive therapy and cognitive behavioral therapy used for suicide prevention.

**Objectives:**

Our objective was to summarize and critically analyze current evidence of effectiveness of SPTIs and no-suicide contracts in suicide prevention.

**Methods:**

We conducted a literature review to compare no-suicide contract to safety-planning interventions in suicide prevention.

**Results:**

Although no-suicide contracts may work for some individuals, there is not enough quantitative evidence to support such contracts as clinically effective tools. A recent meta-analysis has shown that SPTIs were associated with reductions in suicidal behaviors although no effect was identified with frequency of suicidal thoughts *(Nuij et al. (2021, April 30). The British Journal of Psychiatry, 219 (2), 419–426).*

**Conclusions:**

Based on the evidence and straightforward implementation of SPTIs in different clinical settings it may to be a more effective alternative to no-suicide contracts.

**Disclosure of Interest:**

None Declared

